# Proton Density of the Dorsal Root Ganglia in Classical Fabry Disease: MRI Correlates of Small Fibre Neuropathy

**DOI:** 10.3390/biomedicines13061468

**Published:** 2025-06-13

**Authors:** Simon Weiner, Sarah Perleth, Charlotte Schäfer Gómez, Thomas Kampf, Kolja Lau, Florian Hessenauer, György Homola, Peter Nordbeck, Nurcan Üçeyler, Claudia Sommer, Mirko Pham, Magnus Schindehütte

**Affiliations:** 1 Department of Neuroradiology, University Hospital Würzburg, 97080 Würzburg, Germany; 2 Department of Internal Medicine, University Hospital Würzburg, 97080 Würzburg, Germany; 3 Fabry Center for Interdisciplinary Therapy (FAZiT), University Hospital Würzburg, 97080 Würzburg, Germany; 4 Department of Neurology, University Hospital Würzburg, 97080 Würzburg, Germany

**Keywords:** Fabry disease, small fibre neuropathy, dorsal root ganglion, proton density, magnetic resonance imaging

## Abstract

**Background/Objectives:** Fabry disease (FD) is a lysosomal storage disorder often associated with early-onset neuropathic pain, attributed to small fibre neuropathy (SFN). The dorsal root ganglion (DRG) has emerged as a critical site of early pathophysiological involvement in FD, with structural and functional alterations implicated in the development of neuropathic symptoms. This exploratory study introduces DRG proton density (DRG-PD) as a novel MRI-derived biomarker and evaluates its association with SFN. **Methods:** Eighty genetically confirmed FD patients underwent high-resolution 3T MRI with DRG-PD quantification at the lumbosacral levels L5 and S1. DRG-PD was derived from B1-corrected multi-echo spin echo sequences and normalised to cerebrospinal fluid intensity. All patients underwent clinical, biochemical and histological evaluation to determine SFN status. Associations between DRG imaging parameters and clinical variables were analysed using correlation and regression models. Diagnostic performance was evaluated using receiver operating characteristic curve analysis. **Results:** DRG-PD values were significantly increased in patients with classical FD and SFN, demonstrating a large effect size (Cliff’s δ = 0.92) and excellent discriminatory performance (AUC = 0.96). In contrast, DRG volume and T2 relaxation time were not significantly associated with SFN status. DRG-PD remained an independent predictor of SFN in multivariable logistic regression (*p* = 0.019). **Conclusions:** DRG-PD is a non-invasive correlate of SFN in classical FD. It may provide superior diagnostic value compared to existing MRI metrics and reflects proximal ganglionic pathology not captured by distal histological assessments.

## 1. Introduction

Fabry disease (FD) is an X-linked lysosomal storage disorder caused by deficient or absent α-galactosidase A (α-Gal A) activity, resulting in progressive intracellular accumulation of glycosphingolipids, primarily globotriaosylceramide (Gb3). This accumulation affects multiple cell types, including neurons, endothelial cells, and renal epithelial cells [1,2]. One of the earliest and most debilitating clinical manifestations is neuropathic pain, which typically begins in childhood and persists throughout life [3,4]. This pain is generally attributed to small fibre neuropathy (SFN), characterised by selective dysfunction or degeneration of thinly myelinated Aδ fibres and unmyelinated C fibres [4,5,6,7,8].

Recent evidence highlights the dorsal root ganglion (DRG) as a critical anatomical and functional nexus in the pathogenesis of FD-associated neuropathy [9,10]. DRG neurons are one of the earliest sites of glycosphingolipid accumulation and exhibit key pathological features, including endoplasmic reticulum stress, ion channel dysregulation, and ultimately apoptosis [11,12]. Functional studies have revealed increased excitability of DRG neurons, mediated by altered expression of sodium channels (Nav1.7/1.8) and transient receptor potential (TRP) channels, suggesting a mechanistic explanation for pain generation in FD and other SFN conditions [13,14]. More recently, Kaneski et al. developed a CRISPR-based in vitro DRG model of FD that reproduced hallmark features such as increased TRPV1 expression and impaired Gb3 degradation [15].

With recent advances in magnetic resonance imaging (MRI), it is now possible to visualise the DRG in vivo, allowing structural and functional assessment of the neuropathy [16,17,18]. In FD patients with a painful phenotype, DRG enlargement and increased T2 signal intensity have been reported to correlate with both pain severity and FD genotype [19]. Additional studies have implicated impaired DRG perfusion and blood–nerve barrier dysfunction as potential contributors to neuronal stress and altered nociception [20]. Beyond FD, similar DRG imaging findings have been observed in other forms of peripheral neuropathy, highlighting the relevance of DRG-based biomarkers across SFN aetiologies [21,22].

Despite these promising developments, there remains a need for more specific and sensitive DRG imaging metrics. In particular, proton density (PD), an MRI parameter reflecting tissue water content and macromolecular composition, has not yet been systematically investigated in this context. Given the histopathological features of DRG in FD, including lipid accumulation, cytotoxic oedema, and inflammatory infiltrates, PD mapping may provide valuable insights into ganglionic composition in FD [23].

In this study, we investigate DRG proton density (DRG-PD) as a potential biomarker of SFN in FD. By revisiting a previously published imaging dataset using a novel PD quantification approach, we aim to evaluate the diagnostic utility of DRG-PD and its relationship with clinical and histological markers of neuropathy [24]. The focus will be on patients with the classical FD phenotype, who are particularly burdened by neuropathic pain, to determine whether DRG-PD can serve as a sensitive marker of small fibre involvement in this subgroup.

## 2. Materials and Methods

### 2.1. Study Design

This exploratory, hypothesis-generating study builds upon a previously described patient cohort reported by Weiner et al. [24]. We expanded the original analyses by investigating a new imaging parameter, DRG-PD, focusing specifically on its association with SFN.

### 2.2. Study Population

**Figure 1:**
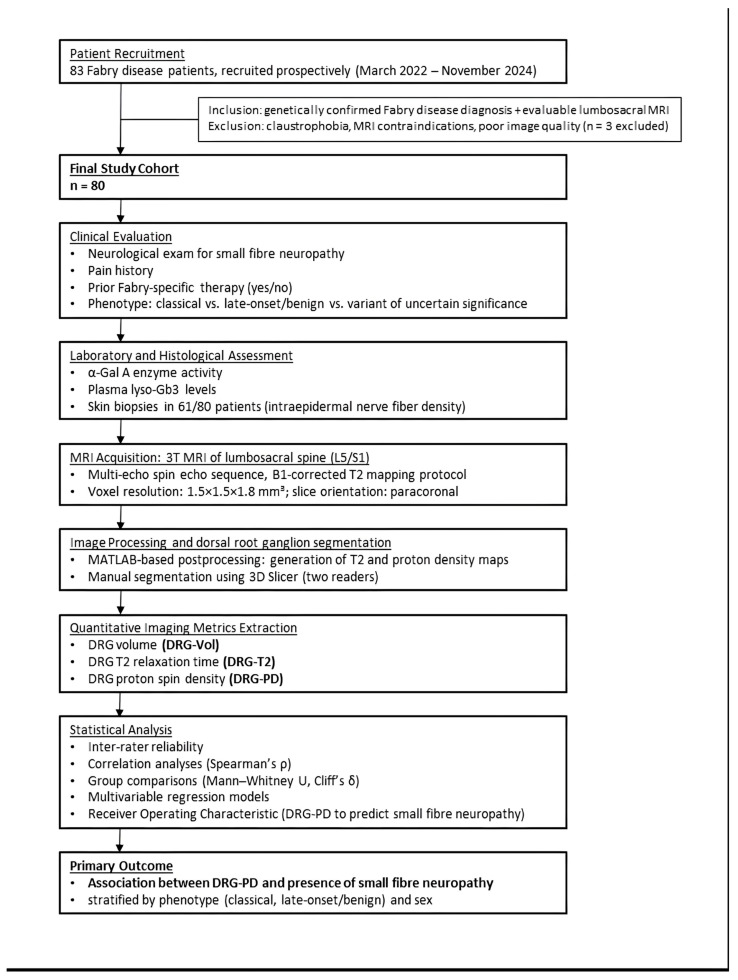
Design of the study. The definitive cohort examined by dorsal root ganglion MRI consisted of 80 patients with a confirmed Fabry disease diagnosis.

Eighty genetically confirmed FD patients were prospectively recruited at the Fabry Center for Interdisciplinary Therapy (FAZiT), University Hospital Würzburg, between March 2022 and November 2024. Inclusion criteria were a confirmed diagnosis and evaluable MRI scans of the lumbosacral region. Three patients were excluded due to insufficient image quality or insufficient DRG delineation. Other exclusion criteria were claustrophobia, contraindications to MRI and lack of informed consent. The study design is summarised as a flowchart in Figure 1.

### 2.3. Clinical Assessment

All participants had a genetically confirmed diagnosis of FD. Mutations were classified as classical, late-onset/benign, or variants of unknown significance (VUS) [25,26,27].

All participants underwent analysis of α-Gal A activity in leukocytes (reference value: 0.4–1.0 nmol/min/mg protein) and plasma lyso-Gb3 levels (reference value: below 0.9 ng/mL).

Skin punch biopsies were taken from the lateral lower leg and the thigh in 61 of the 80 participants. The results were interpreted according to Lauria et al. 2010 [28].

SFN status (SFN-positive or SFN-negative) was established during routine clinical examinations according to Egenolf et al. and Devigili et al. [29,30].

The intensity of the most recent pain episode was assessed using a standardised Numeric Rating Scale (NRS; 0 = no pain, 10 = worst imaginable pain), capturing both average and maximum pain intensity.

### 2.4. MRI Acquisition and Image Post-Processing

All participants underwent MRI on a 3 Tesla scanner (PRISMAfit, Siemens Healthineers, Erlangen, Germany) using a standardised protocol. The imaging protocol included a multi-echo spin echo sequence for T2 mapping of the lumbosacral DRG [31]. Imaging parameters were as follows: echo train length of 10, echo spacing of 15 ms, repetition time of 4.8 s and a flip angle of 180°. The in-plane voxel size was 1.5 × 1.5 mm, with a slice thickness and interslice spacing of 1.8 mm. Images were acquired in a paracoronal orientation to optimally image the DRG at the lumbar (L5) and sacral (S1) levels. These levels were selected as they represent the largest ganglia in the lumbosacral plexus, as well as due to their relevance in lower extremity sensory innervation and their alignment with typical clinical and histological sites of Fabry-associated small fibre pathology.

MR image post-processing was performed using a custom-developed MATLAB (The MathWorks, Inc. (Natick, MA, USA): MATLAB, version R2024b, available at https://www.mathworks.com/) script. The processing workflow included correction for B1 field inhomogeneities and generation of T2 maps from the multi-echo spin echo sequence. Two independent readers manually segmented the DRG at L5 and S1 using 3D Slicer software (The Slicer Community, 2024: 3D Slicer, version 5.6.2, available at https://www.slicer.org/). To minimise partial volume effects, particular attention was given to the exclusion of boundary voxels exhibiting signal artefacts or spill-over into adjacent tissues. These high-quality segmentation masks were then used to calculate quantitative imaging parameters.

### 2.5. Calculation of DRG-PD

DRG-PD was estimated by generating proton density (M0) maps using a multi-echo spin echo sequence, in accordance with the methodology proposed by Neumann et al. [31]. This approach compensates for the influence of stimulated echoes arising from imperfect refocusing pulses, using a voxel-wise correction algorithm based on extended phase graph (EPG) simulations.

First, a mono-exponential fit of the signal decay across echoes was performed for each voxel to obtain preliminary T2 and M0 estimates. Spatial B1 inhomogeneities were accounted for by estimating the effective refocusing flip angle from the ratio of the first two echoes. Based on EPG modelling, a heuristic correction factor was subsequently derived and iteratively applied to refine the M0 values. The final M0 maps were normalised to the cerebrospinal fluid (CSF) signal at the corresponding slice level, yielding a relative measure of DRG proton density. CSF ROIs were manually selected from corrected M0 and T2 maps through thresholding and visual inspection to exclude non-CSF tissue, with final masks verified by a second neuroradiologist. Median DRG M0 values were normalised using the 98th percentile of the CSF M0 within each slice.

### 2.6. Statistical Analysis

All statistical analyses and visualisations were performed using R (The R Foundation for Statistical Computing, 2025: R, version 4.5.0, available at https://www.r-project.org/) and GraphPad Prism (GraphPad Software, 2025: Prism, version 10.4.2, available at https://www.graphpad.com/). A two-sided significance level of *p* < 0.05 was considered statistically significant.

Descriptive statistics for continuous variables were reported as median and interquartile range (IQR), based on non-normal distribution as assessed by the Kolmogorov–Smirnov test. Categorical variables were summarised as absolute numbers and percentages. Group comparisons of demographic, clinical and imaging characteristics between sub-cohorts were performed using the Mann–Whitney U test.

Inter-rater reliability for DRG-Vol, DRG-T2, and DRG-PD was assessed using the intraclass correlation coefficient (ICC; two-way, agreement model), with 95% confidence intervals (CI) and interpretation according to Koo and Li [32].

Spearman’s rank correlation coefficients (ρ) were calculated to examine associations between DRG imaging parameters and continuous clinical or biochemical measures, including age, BMI, lyso-Gb3, α-Gal A activity, and IENFD (lower leg and thigh).

To assess independent associations with DRG-Vol, DRG-T2 and DRG-PD, three separate multivariable linear regression models were constructed, each including the same set of explanatory variables.

For all non-parametric group comparisons, we calculated Cliff’s δ to estimate the effect size and reported its absolute value; the surrounding absolute value bars have been omitted for readability. Interpretation followed standard thresholds [33].

Diagnostic performance of DRG-PD for predicting SFN was assessed using receiver operating characteristic (ROC) curve analyses, conducted separately for classical and late-onset/benign FD phenotypes. The area under the curve (AUC) was calculated for each subgroup, and ROC curves were plotted accordingly.

A multivariable logistic regression model was used to identify predictors of SFN status (binary outcome: yes/no). The model included age, sex, α-Gal A activity, lyso-Gb3 concentration, phenotype (classical vs. other), previous FD-specific therapy (yes/no), DRG-Vol, DRG-T2 and DRG-PD as covariates. Model performance was assessed using Akaike’s information criterion (AIC) and McFadden’s pseudo-R^2^. Results were reported as odds ratios with 95% confidence intervals.

## 3. Results

### 3.1. Study Population Characteristics

A total of 80 patients with FD were included in the final analysis, including 38 males (47.5%) and 42 females (52.5%). Demographic characteristics, clinical data and biochemical parameters are summarised in Table 1, further information on FD genotypes and previous drug treatments can be found in Supplementary Table S1. Descriptive statistics are presented for the entire cohort and stratified by sex.

### 3.2. DRG Imaging Parameters

Median DRG-Vol was 1342.1 mm^3^ (IQR: 1012.6–1610.7) in men and 987.8 mm^3^ (IQR: 769.1–1187.5) in women. The median DRG-T2 was 94.4 ms (IQR: 88.7–108.4) in men and 95.6 ms (IQR: 86.2–102.0) in women. DRG-PD scores had a median of 0.668 arbitrary units (a.u.; IQR: 0.617–0.728) in men and 0.698 a.u. (IQR: 0.653–0.747) in women.

Inter-rater reliability was good to moderate, with ICC of 0.75 (95% CI: 0.63–0.83) for DRG-Vol, 0.76 (95% CI: 0.64–0.84) for DRG-T2, and 0.68 (95% CI: 0.54–0.78) for DRG-PD.

### 3.3. Correlations Between DRG Imaging Parameters and Continuous Clinical Variables

Spearman correlation analyses were performed to examine associations between DRG imaging parameters and continuous clinical and biochemical variables (Table A1), including age, BMI, lyso-Gb3 levels, α-Gal A activity, and IENFD at the lower leg and the thigh.

DRG-Vol showed a significant negative correlation with age (ρ = −0.34, *p* = 0.002) and α-Gal A activity (ρ = −0.32, *p* = 0.004), indicating that higher age and enzymatic activity were associated with smaller DRG volumes. Weak trends towards a negative correlation with BMI and a positive correlation with lyso-Gb3 levels were observed, but did not reach statistical significance.

For DRG-T2, a significant negative correlation with BMI was observed (ρ = −0.32, *p* = 0.004). No other associations for DRG-T2 reached statistical significance.

DRG-PD showed no significant correlations with any of the variables examined. Similarly, IENFD at both biopsy sites showed no significant association with any of the DRG imaging metrics.

### 3.4. DRG Imaging Parameter Associations

Multivariable regression analyses identified several independent determinants of DRG imaging parameters (Table A2). In the model predicting DRG-Vol, male sex was significantly associated with larger volumes (*p* = 0.017). No significant associations were found for age, α-Gal A activity, lyso-Gb3 concentration, classical phenotype, or previous FD-specific therapy.

For DRG-T2, the classical phenotype was significantly associated with higher T2 values (*p* = 0.028), while all other predictors remained nonsignificant.

In the model for DRG-PD, classical phenotype status emerged as a significant predictor of increased PD values (*p* = 0.004). No other covariates showed statistically significant effects in this model.

### 3.5. DRG-PD Is Increased in Classical FD with SFN

Detailed comparisons of DRG-Vol, DRG-T2 and DRG-PD stratified by SFN status are shown in Table 2. Results are shown for the overall cohort, stratified by sex, and further subdivided by FD phenotype (classical vs. benign/late-onset), including sex-specific analyses within each phenotype.

**Figure 2:**
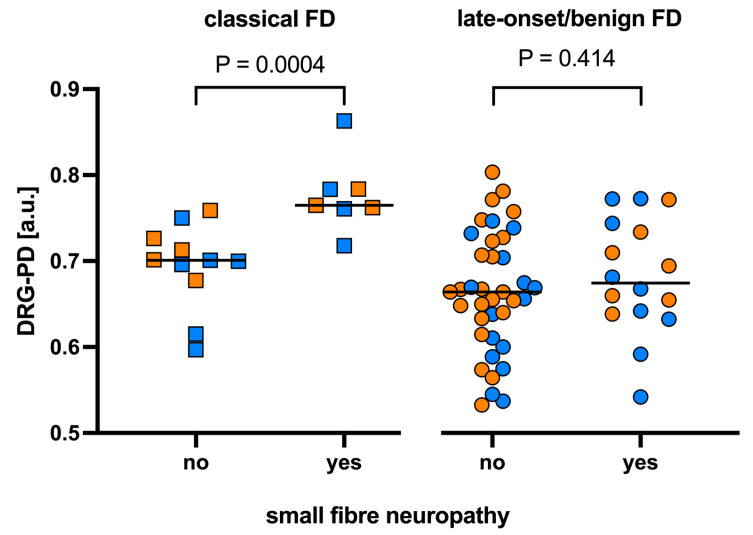
Comparison of proton density of the dorsal root ganglia (DRG-PD) in patients with and without small fibre neuropathy (SFN), stratified by Fabry disease (FD) phenotype (classical and late-onset/benign). Individual data points are colour-coded by shape (blue = male, orange = female). Statistical comparisons were performed using the Mann–Whitney U test; *p*-values are displayed above the boxplots. *Abbreviations:* a.u. = arbitrary units; DRG-PD = dorsal root ganglion proton density.

**Figure 3:**
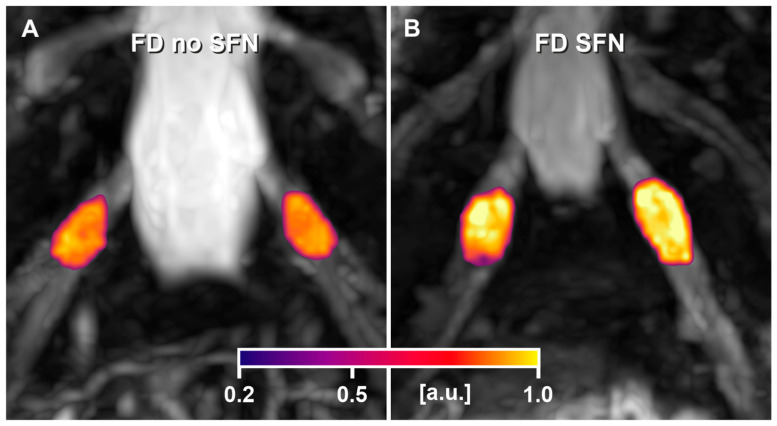
Visualisation of proton density of the dorsal root ganglia (DRG-PD) in two representative patients with the classical Fabry disease (FD) phenotype: one without small fibre neuropathy (SFN) (**A**) and one with SFN (**B**). Voxel-wise DRG-PD heat maps at the bilateral S1 level illustrate increased DRG-PD in the patient with SFN ((**B**), median DRG-PD: 0.863 a.u.) compared to the patient without SFN ((**A**), median DRG-PD: 0.700 a.u.). Abbreviations: a.u. = arbitrary units; DRG-PD = dorsal root ganglion proton density; FD = Fabry disease; S1 = sacral level 1; SFN = small fibre neuropathy.

In the overall cohort, patients with SFN had higher median DRG-PD values compared to those without SFN. In patients with the classical phenotype, this difference was statistically significant (*p* = 0.0004; Figure 2 and Figure 3). The corresponding effect size, quantified by Cliff’s delta, was 0.92 (95% CI: 0.57–0.99), indicating a large effect. These findings remained significant in sex-stratified analyses within the classical phenotype group, both in males (*p* = 0.019) and females (*p* = 0.036). The effect size remained large in both subgroups (men: 0.92, 95% CI: 0.50–0.99; women: 1.00, 95% CI: 0.59–1.00).

In contrast, no significant differences in DRG-PD were observed with respect to SFN status in the benign/late-onset phenotype subgroup.

Reported pain intensity was significantly higher in SFN-positive individuals compared to SFN-negative participants (average pain intensity: 4 [2–5] vs. 2 [0–3], *p* = 0.002, Cliff’s δ = 0.44; maximum pain intensity: 5 [4–7] vs. 3 [0–5], *p* < 0.001, Cliff’s δ = 0.54), with the most pronounced differences observed in those with the classical phenotype (average pain intensity: 5 [0–3] vs. 1 [0–3], *p* = 0.017, Cliff’s δ = 0.70; maximum pain intensity: 6 [3–8] vs. 1 [0–3], *p* = 0.020, Cliff’s δ = 0.69). Among female patients with the classical phenotype, DRG-PD showed strong positive correlations with both average (ρ = 0.896, *p* = 0.003) and maximum (ρ = 0.847, *p* = 0.008) pain intensity. In contrast, no statistically significant correlations were observed between DRG-PD and pain ratings in male patients or in individuals with a late-onset/benign phenotype.

**Figure 4:**
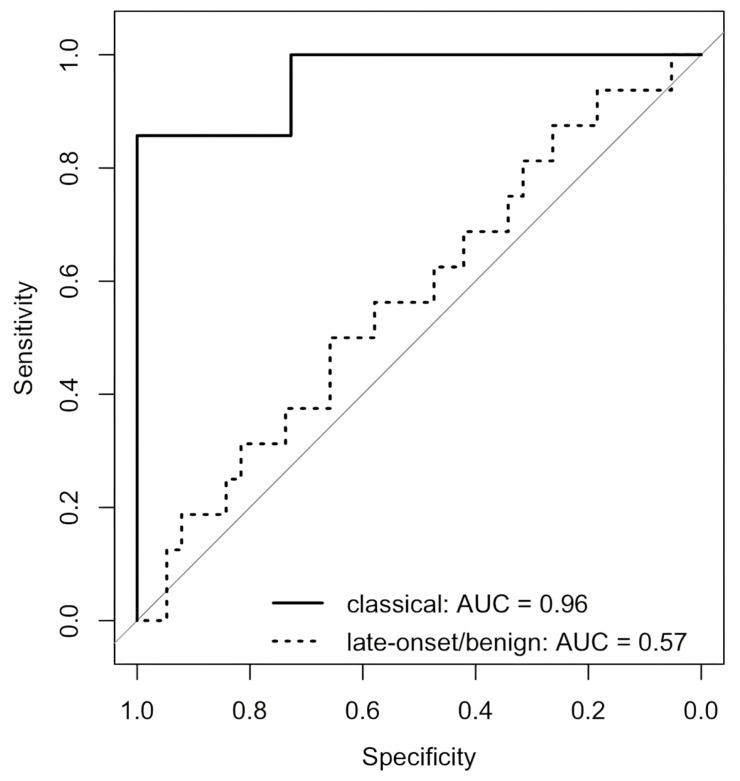
ROC curves for proton density of the dorsal root ganglia (DRG-PD) in predicting small fibre neuropathy (SFN) across Fabry disease (FD) phenotype subgroups. The solid black line represents patients with a classical phenotype (AUC = 0.96), while the dotted black line represents those with a late-onset/benign phenotype (AUC = 0.57). DRG-PD showed excellent discriminatory performance for SFN-status “positive” in the classical phenotype subgroup, whereas discriminatory ability was limited in the late-onset group. The diagonal reference line indicates random classification (AUC = 0.50). SFN status was used as the binary outcome, and DRG-PD values were used as continuous predictors. Abbreviations: AUC = area under the curve; DRG-PD = dorsal root ganglion proton density; FD = Fabry disease; ROC = receiver operating characteristic; SFN = small fibre neuropathy.

Receiver operating characteristic (ROC) analysis (Figure 4) showed excellent discriminatory performance of DRG-PD for the detection of SFN in patients with the classical phenotype (AUC = 0.96). No meaningful discrimination was observed in the benign/late-onset subgroup (AUC = 0.57).

For DRG-Vol and DRG-T2, no statistically significant differences were observed between SFN-positive and SFN-negative patients in any subgroup (Table 2).

### 3.6. Logistic Regression Analysis for SFN Prediction

Multivariable logistic regression analysis was performed to identify independent predictors of SFN (Table 3). The model included age, sex, lyso-Gb3 levels, α-Gal A activity, DRG imaging parameters (DRG-Vol, DRG-T2, DRG-PD), phenotype (classical vs. other), and prior FD-specific therapy.

Significant associations with positive SFN-status were observed for DRG-PD (*p* = 0.019) and prior therapy (*p* = 0.008). No significant associations were found for age, sex, lyso-Gb3, α-Gal A activity, DRG-T2, DRG-Vol or phenotype.

**Figure 5:**
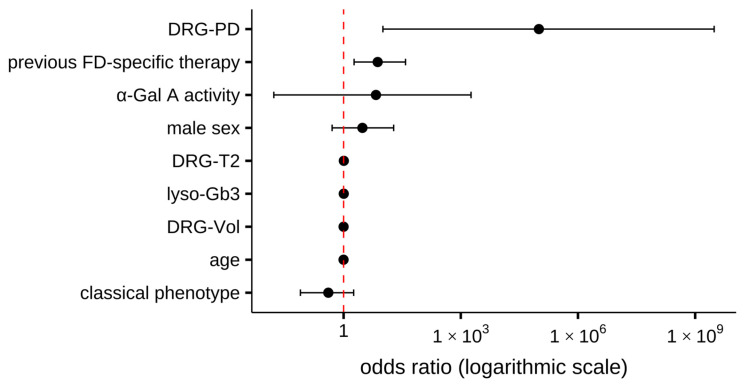
Forest plot of the multivariable logistic regression model predicting the presence of small fibre neuropathy (SFN). Odds ratios with 95% confidence intervals are shown for each covariate included in the model. The vertical dashed line at an odds ratio of 1 indicates no association. Abbreviations: α-Gal A = alpha-galactosidase A; DRG-PD = dorsal root ganglion proton density; DRG-T2 = dorsal root ganglion T2 relaxation time; DRG-Vol = dorsal root ganglion volume; FD = Fabry disease; SFN = small fibre neuropathy.

The model showed an acceptable overall fit, with an Akaike information criterion (AIC) of 100.7 and McFadden’s pseudo-R^2^ of 0.19. Full model estimates, including odds ratios and 95% confidence intervals, are presented in Table 3 and visualised in Figure 5.

## 4. Discussion

This study introduces DRG-PD as a novel MRI-derived parameter with potential utility in the detection of SFN in FD, particularly within the classical phenotype. Building on established imaging markers such as DRG-Vol and DRG-T2, our findings highlight DRG-PD as a phenotype-specific, non-invasive biomarker that reflects proximal sensory ganglion pathology with high discriminative accuracy.

Our results demonstrate a robust association between increased DRG-PD and positive SFN-status in the classical phenotype. This parameter outperformed previous imaging metrics, as neither volume nor T2 values significantly discriminated neuropathy status between subgroups. The excellent diagnostic performance (AUC = 0.96 in the classical phenotype) underscores the clinical value of DRG-PD as a marker of small fibre pathology and reinforces the central role of DRG alterations in FD-associated neuropathy.

These findings extend previous work by Schindehütte et al., who reported increased T2 signal intensity in patients with FD pain, interpreted as a surrogate for inflammation and metabolic dysfunction [31,32]. In contrast, DRG-PD may provide enhanced sensitivity, possibly reflecting macromolecular tissue composition and subtle shifts in water compartments that reflect changes at the cellular level [34,35].

The observed increase in DRG-PD in classical FD is mechanistically consistent with established pathological features of Fabry-associated ganglionopathy. Neuronal accumulation of glycosphingolipids, particularly globotriaosylceramide, impairs lysosomal clearance, induces endoplasmic reticulum stress and alters ion channel dynamics, potentially leading to hyperexcitability and small diameter fibre degeneration [33]. These mechanisms are supported by experimental studies, including transcriptional profiling of ganglia from α-galactosidase A-deficient mice, which revealed dysregulation of pathways involved in lysosomal metabolism, G protein-coupled receptor signalling and immune modulation [36]. Altered ion channel function, particularly involving Nav1.7, Nav1.8 and TRPV1, further contributes to reduced activation thresholds and increased nociceptor excitability [33]. In vitro models using CRISPR-engineered neuronal cells have supported these findings, demonstrating impaired globotriaosylceramide degradation and TRPV1 upregulation consistent with sensitisation mechanisms [34]. These changes are expected to elevate tissue water content and disrupt macromolecular architecture, key determinants of MR proton density. As such, DRG-PD may serve as a functional in vivo correlate of these proximal, ganglion-specific pathological processes.

Imaging studies in FD have also suggested involvement of the DRG. Godel et al. reported ganglion enlargement and reduced perfusion, particularly within neuronal cell body-rich regions, reflecting inflammatory and vascular changes [37]. In the context of bortezomib-induced neuropathy, Zhou et al. described increased T2 signal intensities associated with decreased IENFD, while DRG volume remained unchanged, similar to our current findings [38].

Notably, none of the imaging parameters, including DRG-PD, showed significant correlations with IENFD in our cohort. This suggests that DRG-PD may reflect proximal neuronal pathology that is not reflected in distal axon loss, consistent with a non-length-dependent pattern of neuropathy as described by Gorson et al. and Gemignani et al., which is often driven by DRG impairment rather than distal axonopathy [6,39].

Although DRG imaging markers were not significantly associated with self-reported pain intensity in most subgroups, a notable exception was observed in female patients with the classical FD phenotype, where DRG-PD demonstrated strong correlations with both average and maximum pain ratings. While based on a limited sample size, this finding points to a potentially meaningful relationship between ganglionic proton density and the subjective pain experience in this subgroup. The absence of significant DRG-PD alterations in patients with non-classical (late-onset or benign) FD phenotypes likely reflects the underlying biochemical and clinical heterogeneity of this subgroup. These individuals typically retain partial α-Gal A activity, resulting in lower levels of neuronal substrate accumulation and less severe DRG involvement. As a result, structural and compositional changes at the ganglion level may not reach the threshold required to generate measurable alterations in proton density. In addition, the overall burden of SFN is reduced in this phenotype, further limiting the detectability of DRG-PD as a diagnostic marker. Beyond FD, DRG imaging is gaining relevance in other neuropathies, including immune-mediated and idiopathic forms, where it may serve as a tool for diagnosis, stratification and even therapeutic guidance. In particular, targeted interventions such as neuromodulation of the dorsal root ganglia are being evaluated [40,41].

Although we observed that changes in DRG-PD correlated with both SFN status and pain scores in only a small subgroup of female FD patients, this may be because women experience delayed or more prolonged damage to C-fibres. This contrasts with male FD patients, who typically experience pain at an earlier age. A previous study considered the possibility that secondary central sensitisation contributes to the development of non-ganglionic pain in male FD patients [42].

Although DRG-PD shows promise as a disease-specific imaging biomarker, several limitations must be acknowledged. First, the cross-sectional and exploratory design limits conclusions regarding temporal evolution or causality. Longitudinal studies are needed to determine whether DRG-PD responds to therapeutic intervention and can serve as a marker for disease monitoring. Second, the relatively small sample size, especially within the classical phenotype, may have limited statistical power and generalisability. The limited sample size may have reduced statistical power, potentially obscuring clinically relevant effects. Further studies are needed that include larger groups of patients with classical FD to confirm the observed trends and strengthen the validity of these findings. Third, the absence of a healthy control group limits the interpretation of absolute DRG-PD values, since it prevents the establishment of normative reference ranges. Consequently, our findings should be interpreted in the context of intra-cohort comparisons. It will be essential for future studies to include age- and sex-matched healthy controls in order to determine the specificity of the DRG-PD alterations observed in FD. Moreover, due to the small size of the DRG and its close anatomical proximity to the CSF and vertebral structures, partial volume effects may influence signal estimation, particularly at tissue interfaces. Although manual segmentation and high-resolution imaging helped mitigate these effects, minor inaccuracies in ROI delineation or spatial registration could still affect the accuracy of PD estimates and must be considered when interpreting the results. Finally, monitoring DRG-PD longitudinally presents challenges, including scanner-to-scanner variability and the need for standardised protocols. Furthermore, practice effects may influence longitudinal pain assessments. Objective sensory testing and standardised rater training are therefore necessary to ensure that any observed changes in PD genuinely reflect disease progression.

## 5. Conclusions

In conclusion, our findings establish DRG-PD as a sensitive and non-invasive imaging correlate for SFN in the classical FD phenotype. By targeting the DRG, an anatomically and functionally central structure in the pathogenesis of SFN, DRG-PD may serve as a proximal and disease-relevant marker of small fibre involvement; however, its value for longitudinal monitoring remains to be determined in future studies.

Prospective longitudinal studies incorporating diverse neuropathy aetiologies and therapeutic interventions are needed to establish whether DRG-PD is responsive to disease-modifying treatments and can capture pathological progression over time.

## Figures and Tables

**Table 1:** Demographic characteristics of the study cohort (n = 80) with FD, shown as median values with IQR or count (percentage), respectively. Data are additionally stratified by sex (male and female).

Parameter	All (n = 80) [Median (IQR) or Count (Percentage)]	Men (n = 38) [Median (IQR) or Count (Percentage)]	Women (n = 42) [Median (IQR) or Count (Percentage)]
Age [years]	42.0 (33.0–57.0)	36.0 (29.2–44.0)	52.0 (35.2–62.8)
Height [cm]	168.0 (165.0–178.0)	178.0 (173.5–183.8)	165.0 (163.0–167.8)
Weight [kg]	71.5 (62.5–85.2)	80.0 (70.2–89.0)	67.0 (58.0–78.8)
BMI [kg/m^2^]	24.5 (21.9–27.8)	24.7 (23.0–26.4)	24.0 (21.6–28.9)
α-Gal A [nmol/min/mg protein]	0.17 (0.04–0.29)	0.04 (0.04–0.06)	0.28 (0.23–0.39)
lyso-Gb3 [ng/mL]	11.1 (2.0–22.6)	20.1 (6.9–60.8)	4.2 (1.1–13.8)
IENFD, lower leg [mm⁻¹]	4.2 (2.4–7.0)	2.8 (0.8–5.1)	5.2 (3.5–8.5)
IENFD, thigh [mm⁻¹]	18.3 (13.5–26.2)	16.2 (12.7–20.4)	21.4 (16.7–32.6)
Previous FD-specific treatment	47 (58.8%)	28 (73.7%)	19 (45.2%)
Previous cerebrovascular event	13 (16.2%)	7 (18.4%)	6 (14.3%)
History of FD pain	48 (60%)	25 (65.8%)	23 (54.8%)
Average pain intensity (NRS)	2 (0–5)	3 (0–4)	2 (0–5)
Maximum pain intensity (NRS)	3 (0–6)	3 (0–6)	3 (0–6)
SFN	25 (31.2%)	15 (39.5%)	10 (23.8%)
Previous antineuropathic treatment	18 (22.5%)	10 (26.3%)	8 (19%)
Classical phenotype	18 (22.5%)	10 (26.3%)	8 (19%)
Late-onset or benign phenotype	54 (67.5%)	24 (63.2%)	30 (71.4%)
VUS	8 (10%)	4 (10.5%)	4 (9.5%)

Abbreviations: α-Gal A = alpha-galactosidase A; BMI = body mass index; FD = Fabry disease; IENFD = intraepidermal nerve fibre density; IQR = interquartile range; lyso-Gb3 = globotriaosylsphingosine; NRS = Numeric Rating Scale; SFN = small fibre neuropathy; VUS = variant of uncertain significance.

**Table 2:** Comparison of dorsal root ganglion (DRG) imaging parameters between patients with and without small fibre neuropathy (SFN). Results are presented as median values with interquartile ranges (IQR), including *p*-values for group comparisons (Mann–Whitney U test).

Subcohort	DRG-Parameter	[n]	SFN-Positive [Median (IQR)]	[n]	SFN-Negative [Median (IQR)]	*p*-Value	Cliff’s δ
All	DRG-Vol [mm^3^]	25	1105.2 (784.4–1390.9)	55	1089.1 (884.3–1377.1)	0.934	0.01
All	DRG-T2 [ms]	25	96.4 (89.9–107.1)	55	94.9 (85.1–102.3)	0.461	0.10
All	DRG-PD [a.u.]	25	0.710 (0.655–0.765)	55	0.667 (0.636–0.725)	0.063	0.26
m	DRG-Vol [mm^3^]	15	1302.0 (914.2–1646.1)	23	1354.5 (1080.4–1561.5)	0.595	0.11
m	DRG-T2 [ms]	15	99.3 (90.0–107.9)	23	92.5 (82.8–105.6)	0.378	0.17
m	DRG-PD [a.u.]	15	0.681 (0.637–0.766)	23	0.656 (0.605–0.700)	0.114	0.31
f	DRG-Vol [mm^3^]	10	921.5 (606.5–1117.2)	32	992.2 (794.2–1208.0)	0.631	0.11
f	DRG-T2 [ms]	10	94.3 (87.3–102.6)	32	95.6 (86.0–101.8)	0.965	0.01
f	DRG-PD [a.u.]	10	0.722 (0.668–0.764)	32	0.672 (0.649–0.732)	0.202	0.28
Classical	DRG-Vol [mm^3^]	7	1003.1 (809.9–1113.2)	11	1181.0 (896.7–1563.0)	0.319	0.30
Classical	DRG-T2 [ms]	7	104.4 (93.5–113.9)	11	102.1 (92.0–117.9)	1.0	0.01
Classical	DRG-PD [a.u.]	7	0.765 (0.761–0.784)	11	0.701 (0.687–0.720)	0.0004	0.92
Classical; m	DRG-Vol [mm^3^]	4	809.9 (760.7–1043.6)	6	1314.4 (944.8–1639.2)	0.352	0.42
Classical; m	DRG-T2 [ms]	4	98.8 (89.8–110.8)	6	102.3 (91.8–120.9)	0.610	0.25
Classical; m	DRG-PD [a.u.]	4	0.772 (0.750–0.803)	6	0.698 (0.635–0.701)	0.019	0.92
Classical; f	DRG-Vol [mm^3^]	3	1105.2 (1054.1–1113.2)	5	1181.0 (896.7–1262.6)	0.786	0.20
Classical; f	DRG-T2 [ms]	3	104.4 (100.4–112.6)	5	102.1 (96.3–102.7)	0.571	0.33
Classical; f	DRG-PD [a.u.]	3	0.765 (0.763–0.774)	5	0.713 (0.702–0.726)	0.036	1.00
Benign/late-onset	DRG-Vol [mm^3^]	16	1156.2 (750.1–1449.3)	38	1037.4 (852.2–1348.3)	0.673	0.08
Benign/late-onset	DRG-T2 [ms]	16	92.3 (89.7–99.8)	38	94.1 (83.8–101.4)	0.842	0.04
Benign/late-onset	DRG-PD [a.u.]	16	0.674 (0.641–0.736)	38	0.664 (0.619–0.719)	0.414	0.15
Benign/late-onset; m	DRG-Vol [mm^3^]	9	1390.9 (1074.5–1697.1)	15	1329.7 (1122.7–1525.1)	0.861	0.05
Benign/late-onset; m	DRG-T2 [ms]	9	99.3 (90.1–100.4)	15	91.8 (79.5–100.8)	0.245	0.30
Benign/late-onset; m	DRG-PD [a.u.]	9	0.668 (0.632–0.744)	15	0.656 (0.594–0.689)	0.446	0.20
Benign/late-onset; f	DRG-Vol [mm^3^]	7	768.4 (551.9–1038.8)	23	962.3 (580.3–1044.7)	0.737	0.09
Benign/late-onset; f	DRG-T2 [ms]	7	89.9 (81.6–94.7)	23	94.4 (88.7–101.3)	0.327	0.26
Benign/late-onset; f	DRG-PD [a.u.]	7	0.694 (0.657–0.722)	23	0.664 (0.644–0.725)	0.598	0.14

Abbreviations: DRG = dorsal root ganglion; DRG-PD = dorsal root ganglion proton density; DRG-T2 = dorsal root ganglion T2 relaxation time; DRG-Vol = dorsal root ganglion volume; f = female; m = male; IQR = interquartile range; SFN = small fibre neuropathy.

**Table 3:** Results of logistic regression analysis examining predictors of SFN status in patients with Fabry disease. Model fit statistics: Akaike information criterion = 100.68, residual deviance = 80.68, pseudo R^2^ (McFadden) = 0.19, likelihood ratio test *p* = 0.028 (n = 80).

Variable	Estimate	Std. Error	z-Value	*p*-Value
(Intercept)	−10.640	3.986	−2.669	0.008
Age	−0.004	0.022	−0.182	0.855
Sex (male)	1.108	0.913	1.214	0.225
lysoGb3	0.009	0.007	1.195	0.232
α-Gal A enzyme activity	1.907	2.898	0.658	0.511
DRG-PD	11.510	4.914	2.343	0.019
DRG-T2	0.012	0.020	0.631	0.528
DRG-Vol	−0.0012	0.0007	−1.646	0.100
Phenotype (classical)	−0.902	0.791	−1.141	0.254
Previous FD-specific treatment (yes)	2.008	0.762	2.635	0.008

Abbreviations: α-Gal A = alpha-galactosidase A; DRG = dorsal root ganglion; DRG-PD = dorsal root ganglion proton density; DRG-T2 = dorsal root ganglion T2 relaxation time; DRG-Vol = dorsal root ganglion volume; FD = Fabry disease; lysoGb3 = globotriaosylsphingosine; SFN = small fibre neuropathy.

## Data Availability

The patient cohort has been described previously (https://doi.org/10.3390/biomedicines13030592). The datasets generated and analysed during the current study are not publicly available due to data privacy reasons, but are available from the corresponding author upon reasonable request.

## Supplementary Materials

The following supporting information can be downloaded at: https://www.mdpi.com/article/10.3390/biomedicines13061468/s1, Table S1: Cohort Characteristics.

## Abbreviations

The following abbreviations are used in this manuscript:

AUC	area under the curve
a.u.	arbitrary units
α-Gal A	alpha-galactosidase A
BMI	body mass index
DRG	dorsal root ganglion
DRG-PD	dorsal root ganglion proton density
DRG-T2	dorsal root ganglion T2 relaxation time
DRG-Vol	dorsal root ganglion volume
FD	Fabry disease
f	female
Gb3	globotriaosylceramide
GLA	galactosidase A gene
IENFD	intraepidermal nerve fibre density
IQR	interquartile range
lysoGb3	globotriaosylsphingosine
m	male
NRS PD	Numeric Rating Scale proton density
PNS	peripheral nervous system
ROC	receiver operating characteristic
SFN	small fibre neuropathy
VUS	variant of uncertain significance
ρ	Spearman’s rank correlation coefficient

## Appendix A

**Table A1:** Spearman correlation coefficients (ρ) and associated *p*-values describing correlations between clinical characteristics (age, BMI, lysoGb3, enzyme activity, and IENFD) and DRG MRI parameters.

DRG Parameter	Variable	Spearman’s ρ	*p*-Value
DRG-Vol	Age	−0.339	0.002
DRG-Vol	BMI	−0.211	0.061
DRG-Vol	lysoGb3	0.206	0.067
DRG-Vol	α-Gal A enzyme activity	−0.317	0.004
DRG-Vol	IENFD, lower leg	−0.050	0.704
DRG-Vol	IENFD, thigh	−0.015	0.911
DRG-Vol	Average pain intensity	−0.013	0.915
DRG-Vol	Maximum pain intensity	−0.023	0.844
DRG-T2	age	−0.134	0.236
DRG-T2	BMI	−0.318	0.004
DRG-T2	lysoGb3	0.160	0.156
DRG-T2	α-Gal A enzyme activity	−0.104	0.359
DRG-T2	IENFD, lower leg	0.028	0.830
DRG-T2	IENFD, thigh	−0.023	0.860
DRG-T2	Average pain intensity	0.052	0.662
DRG-T2	Maximum pain intensity	0.163	0.165
DRG-PD	Age	0.082	0.468
DRG-PD	BMI	−0.061	0.590
DRG-PD	lysoGb3	0.039	0.729
DRG-PD	α-Gal A enzyme activity	0.109	0.335
DRG-PD	IENFD, lower leg	−0.076	0.563
DRG-PD	IENFD, thigh	−0.122	0.348
DRG-PD	Average pain intensity	−0.017	0.883
DRG-PD	Maximum pain intensity	0.006	0.957

Abbreviations: α-Gal A = alpha-galactosidase A; BMI = body mass index; DRG = dorsal root ganglion; DRG-PD = dorsal root ganglion proton density; DRG-T2 = dorsal root ganglion T2 relaxation time; DRG-Vol = dorsal root ganglion volume; IENFD = intraepidermal nerve fibre density; lysoGb3 = globotriaosylsphingosine; ρ = Spearman’s rank correlation coefficient.

**Table A2:** Results of regression analyses examining associations between clinical variables (age, enzyme activity, lysoGb3 levels, classical phenotype, male sex, and previous FD-specific therapy) and dorsal root ganglion MRI parameters. Estimates and corresponding *p*-values are provided.

Variable	Estimate (DRG-Vol)	*p*-Value (DRG-Vol)	Estimate (DRG-T2)	*p*-Value (DRG-T2)	Estimate (DRG-PD)	*p*-Value (DRG-PD)
(Intercept)	1188.455	<0.001	104.793	<0.001	0.676	<0.001
Age	−4.33	0.217	−0.112	0.345	0.0	0.914
Enzyme activity	−56.402	0.905	−12.973	0.423	0.017	0.818
lysoGb3	0.005	0.997	−0.037	0.390	0.0	0.686
Phenotype (classical)	−53.446	0.669	9.451	0.028	0.059	0.004
Sex (male)	346.579	0.017	0.142	0.977	−0.024	0.292
Previous FD-specific therapy (yes)	12.806	0.905	−3.569	0.328	−0.007	0.698

Abbreviations: DRG = dorsal root ganglion; DRG-PD = dorsal root ganglion proton density; DRG-T2 = dorsal root ganglion T2 relaxation time; DRG-Vol = dorsal root ganglion volume; FD = Fabry disease; lysoGb3 = globotriaosylsphingosine.
