# Supplementary material for: Proton Density of the Dorsal Root Ganglia in Classical Fabry Disease: MRI Correlates of Small Fibre Neuropathy
Source: Biomedicines. 2025 Jun 13;13(6):1468. doi: 10.3390/biomedicines13061468 (PMC12191292; doi:10.3390/biomedicines13061468)
Supplement: Supplementary file 1 [file biomedicines-13-01468-s001.zip › biomedicines-3648045-supplementary.pdf]

Supplementary Table S1. Cohort Characteristics.

id	sex	age [years]	alpha-galactosidase mutation	type of mutation	significance	previous FD-specific therapy	enzyme activity [nmol/min/mg protein]	lyso-Gb3 level [ng/mL]	previous analgesic or antineuropathic therapy
**1**	m	28	Intron 3, IVS2-81..-77 del + IVS4-16A>G, IVS6-22 C>T	Splice/In-frame	VUS	no	0.57	0.7	no
**2**	m	62	Transition c.644 A>G // p.N215S	Missense	late-onset/benign	no	0.05	3.0	no
**3**	f	39	c.718_719del // p.K240Efs*9	Frameshift	classic	ERT	0.17	22.8	yes
**4**	m	34	c.350T>G // p.I117S	Missense	late-onset/benign	study drug (Lucerastat)	0.06	79.9	no
**5**	f	53	c.612G>T // p.W204C	Missense	late-onset/benign	ERT	0.15	11.9	no
**6**	f	65	c.937G>T //p.D313Y	Missense	late-onset/benign	no	0.40	1.0	no
**7**	m	57	c.1021G>A // p.E341K	Missense	late-onset/benign	ERT	0.02	22.5	no
**8**	f	52	c.658C>T // p.Arg220*	Nonsense	classic	ERT	0.27	18.5	no
**9**	m	37	c.404C>T // p.A135V	Missense	late-onset/benign	chaperone	0.04	138.0	yes
**10**	f	72	c.644A>G // p.N215S	Missense	late-onset/benign	no	0.40	1.5	yes
**11**	m	40	c.1091_1092 delCT // p.365fs*9	Frameshift	classic	ERT	0.05	94.7	yes
**12**	m	24	c.994dup // p.R332Kfs*7	Frameshift	classic	ERT	0.02	39.6	no
**13**	m	71	c.644A>G // p.N215S	Missense	late-onset/benign	chaperone	0.05	8.1	no
**14**	f	57	c.352C>T // p.Arg118Cys	Missense	late-onset/benign	no	0.45	0.6	no
**15**	f	64	Transition c.644 A>G // p.N215S	Missense	late-onset/benign	no	0.37	1.1	no
**16**	f	67	c.973G>A // p.G325S	Missense	late-onset/benign	chaperone	0.02	3.8	yes
**17**	m	21	c.124A>G // p.M42V	Missense	late-onset/benign	no	0.05	12.8	no
**18**	f	47	c.1250T>C // p.L417P	Missense	late-onset/benign	ERT	0.41	13.3	no
**19**	m	44	c.1095T>A // p.Tyr365Ter	Nonsense	classic	no	0.04	184.0	yes
**20**	m	34	Transition c.644 A>G // p.N215S	Missense	late-onset/benign	no	0.05	7.1	no
**21**	m	44	c.426C>A // p.C142X	Nonsense	classic	ERT	0.04	62.4	yes
**22**	m	36	c.515G>A // p.C172Y	Missense	late-onset/benign	no	0.04	193.0	no
**23**	f	59	c.137A>G // p.H46R	Missense	late-onset/benign	ERT	0.41	12.2	yes
**24**	f	49	c.994dup // p.R332Kfs*7	Frameshift	classic	ERT	0.20	11.0	no
**25**	f	43	c.119C>T // p.P40L	Missense	late-onset/benign	no	0.27	16.8	no
**26**	m	26	c.424T>C // p.C142R	Missense	late-onset/benign	ERT	0.04	52.6	yes

**27**	f	49	c.427G>A // p.A143T	Missense	late-onset/benign	no	0.27	1.1	no
**28**	m	27	c.363delT // A121fs*8	Frameshift	classic	ERT	0.05	98.7	yes
**29**	m	29	c.363delT // p.A121fs*8	Frameshift	classic	ERT	0.05	41.4	no
**30**	f	62	c.934C>T // p.Q312X	Nonsense	classic	ERT	0.05	16.2	no
**31**	f	36	c.934C>T // p.Q312X	Nonsense	classic	no	0.26	25.8	no
**32**	f	60	c.404C>T // p.A135V	Missense	late-onset/benign	chaperone	0.29	21.4	no
**33**	f	33	c.404C>T // p.A135V	Missense	late-onset/benign	chaperone	0.25	20.9	no
**34**	f	32	c.335G>A // p.R112H	Missense	late-onset/benign	ERT	0.50	0.8	no
**35**	m	30	c.1072_1074del // p.E358del	In-frame	VUS	no	0.02	202.0	yes
**36**	f	69	IVS2-81-77CAGCC	Splice	VUS	no	0.45	0.8	yes
**37**	f	43	c.126G>A // p.Met42Ile	Missense	late-onset/benign	ERT	0.25	7.8	no
**38**	m	33	c.1244T>G // p.L415R	Missense	late-onset/benign	ERT	0.20	11.2	no
**39**	m	40	c.386T>C // p.L129P	Missense	late-onset/benign	ERT	0.03	51.9	no
**40**	f	80	c.427G>A // p.A143T	Missense	late-onset/benign	ERT	0.37	1.4	no
**41**	m	35	c.973G>A // p.G325S	Missense	late-onset/benign	chaperone	0.06	17.4	no
**42**	m	60	c.644A>G // p.N215S	Missense	late-onset/benign	chaperone	0.23	6.8	no
**43**	f	24	c.1209_1211del AAG	In-frame	VUS	no	0.16	14.5	yes
**44**	m	29	c.416A>G // p.N139S	Missense	late-onset/benign	dual	0.04	11.5	no
**45**	m	38	c.72G>A // p.W24X	Nonsense	classic	ERT	0.04	160.0	no
**46**	m	32	c.486G>T // p.W162C	Missense	late-onset/benign	dual	0.04	17.7	yes
**47**	f	54	c.1072_1074del // p.E358del	In-frame	VUS	ERT	0.27	16.1	no
**48**	f	72	c.1091_1092delCT	Frameshift	classic	no	0.29	4.5	yes
**49**	m	49	c.427G>A // p.A143T	Missense	late-onset/benign	no	0.19	0.8	no
**50**	m	44	c.486G>T // p.W162C	Missense	late-onset/benign	ERT	0.02	7.6	no
**51**	f	32	Transition c.644 A>G // p.N215S	Missense	late-onset/benign	no	0.20	2.9	yes
**52**	f	18	c.119C>T // p.40L	Missense	late-onset/benign	no	0.33	13.2	no
**53**	f	31	c.644A>G // p.N215S	Missense	late-onset/benign	no	0.22	1.4	yes
**54**	m	45	c.1000-10G>A	Splice	VUS	ERT	0.04	30.8	yes
**55**	f	52	c.427G>A // p.A143T	Missense	late-onset/benign	no	0.41	0.7	no
**56**	f	60	c.427G>A // p.A143T	Missense	late-onset/benign	no	0.29	0.8	yes
**57**	m	27	c.658 C>T // R220X	Nonsense	classic	no	0.03	137.0	no
**58**	m	39	c.508G>A // p.D170N	Missense	late-onset/benign	ERT	0.02	12.1	no

**59**	f	46	c.427G>A // p.A143T	Missense	late-onset/benign	no	0.27	0.9	yes
**60**	m	43	c.427G>A // p.A143T	Missense	late-onset/benign	no	0.06	0.8	yes
**61**	f	33	c.902G>A // p.R301Q	Missense	late-onset/benign	ERT	0.24	2.5	no
**62**	m	36	c.757del // p.I253Lfs*16	Frameshift	classic	Replagal	0.04	56.1	no
**63**	f	27	Transition c.644 A>G // p.N215S	Missense	late-onset/benign	no	0.58	0.7	yes
**64**	m	63	c.644 A>G // p.N215S	Missense	late-onset/benign	chaperone	0.06	3.1	no
**65**	f	41	c.1000-1G>A	Splice	VUS	ERT	0.15	5.6	no
**66**	f	64	c.994dup // p.R332Kfs*7	Frameshift	classic	ERT	0.31	9.0	yes
**67**	f	63	c.416A>G // p.N139S	Missense	late-onset/benign	chaperone	0.14	5.2	no
**68**	f	57	c.973G>A // p.G325S	Missense	late-onset/benign	no	0.35	4.0	no
**69**	f	24	c.644A>G // p.N215S	Missense	late-onset/benign	no	0.24	1.3	no
**70**	m	53	c.644A>G // p.N215S	Missense	late-onset/benign	ERT	0.04	4.8	yes
**71**	f	56	Transition c.644 A>G // p.N215S	Missense	late-onset/benign	no	0.45	1.2	yes
**72**	m	39	c.1067G>A // p.R356Q	Missense	late-onset/benign	chaperone	0.18	1.5	yes
**73**	f	34	c.708G>C // p.W236C	Missense	late-onset/benign	no	0.29	17.8	no
**74**	m	25	c.963_964delinsCA // p.Q321_D322delins HN	In-frame	VUS	ERT	0.03	29.5	no
**75**	m	33	c.993_994 ins A fs*338	Frameshift	classic	ERT	0.03	41.0	yes
**76**	f	35	c.1069C>T // p.Q357X	Nonsense	classic	no	0.15	13.9	no
**77**	m	36	c.644A>G // p.N215S	Missense	late-onset/benign	chaperone	0.11	2.1	yes
**78**	f	63	c.644A>G // p.N215S	Missense	late-onset/benign	chaperone	0.39	2.3	yes
**79**	f	64	c.1184G>C // p.G395A	Missense	late-onset/benign	no	0.30	0.8	yes
**80**	m	24	Transition c.644 A>G // p.N215S	Missense	late-onset/benign	chaperone	0.05	5.1	no

Abbreviations: ERT = enzyme replacement therapy; lyso-Gb3 = Globotriaosylsphingosine; VUS = variant of unknown significance.
